# Limb blastema formation: How much do we know at a genetic and epigenetic level?

**DOI:** 10.1016/j.jbc.2022.102858

**Published:** 2022-12-31

**Authors:** Sangwon Min, Jessica L. Whited

**Affiliations:** Department of Stem Cell and Regenerative Biology, Harvard University, Cambridge, Massachusetts, USA

**Keywords:** regeneration, stem cells, epigenetics, morphogenesis, cell signaling, AEC, apical epidermal cap, ALM, accessory limb model, DNMT, DNA methyltransferase, EMT, epithelial-to-mesenchymal transition, FGF, fibroblast growth factor, HDAC, histone deacetylase, TGF-β, transforming growth factor-beta

## Abstract

Regeneration of missing body parts is an incredible ability which is present in a wide number of species. However, this regenerative capability varies among different organisms. Urodeles (salamanders) are able to completely regenerate limbs after amputation through the essential process of blastema formation. The blastema is a collection of relatively undifferentiated progenitor cells that proliferate and repattern to form the internal tissues of a regenerated limb. Understanding blastema formation in salamanders may enable comparative studies with other animals, including mammals, with more limited regenerative abilities and may inspire future therapeutic approaches in humans. This review focuses on the current state of knowledge about how limb blastemas form in salamanders, highlighting both the possible roles of epigenetic controls in this process as well as limitations to scientific understanding that present opportunities for research.

Regeneration is a process of restoring the lost organs, tissue, appendages, or large body parts. The regenerative capacity varies among different species spanning from flatworms, such as planaria, showing a fascinating ability to regenerate body parts including new heads, tails, or even entire organism from a small body fragment (1, 2), to vertebrates, including salamanders as well as teleost fish, such as zebrafish, both of which can fully restore many body parts including limbs and fins (3, 4). Unlike these species, humans have more limited natural abilities to regenerate complex body parts and can only regenerate a few, such as liver, ribs, and digit tips (5, 6, 7, 8, 9, 10). In addition to such limited regenerative ability, humans often exhibit incomplete regeneration; for example, the newly-formed digit tips have cosmetic deformities and/or other physiological limitations including numbness or hypersensitivity (8, 9, 10). Loss of larger area, such as a full digit, a hand, a foot, or the entire limb or organ, results in an inability to regenerate and, thus, requires allogenic transplant or use of prosthetics as treatment. Such inability to regenerate in humans has sparked a profound interest in several complementary biomedical fields towards understanding and, ultimately, stimulating regeneration. Among them, understanding limb regeneration in salamanders has held longstanding scientific intrigue over the last several centuries. The advent of molecular genetic tools operational in salamanders (11, 12, 13, 14, 15, 16, 17, 18, 19, 20, 21) is now enabling a more solid and refined understanding of natural limb regeneration.

In salamanders, successful limb regeneration can be divided into two major phases: (1) formation of a blastema that resembles the early embryonic limb bud, with a few key differences which include innervation-dependent blastema formation (22, 23, 24) and (2) blastema-mediated redevelopment, which involves growth and redifferentiation (Fig. 1). Upon amputation, a specialized wound epidermis is formed *via* cell migration (25) within few hours after injury. Wound epidermal cells become innervated and this transient tissue is thickened with continued migration of dermal cells beneath the wound epidermis and cellular proliferation (26, 27, 28). Subsequently, a visible blastema is formed at the end of the stump, directly beneath the wound epidermis. The blastema cells undergo several rounds of expansion until the blastema acquires a cone shape that then broadens and initiates differentiation. These cells grow in a proximal-to-distal direction obeying the rule of distal transformation, in which tissues can only regenerate the structures distal to the amputation plane (29, 30), and the new tissues reconnect to the stump. The patterning and growth processes following blastema formation largely recapitulates the behavior of a limb bud that forms during embryonic limb development (27, 31).

**Figure 1:**
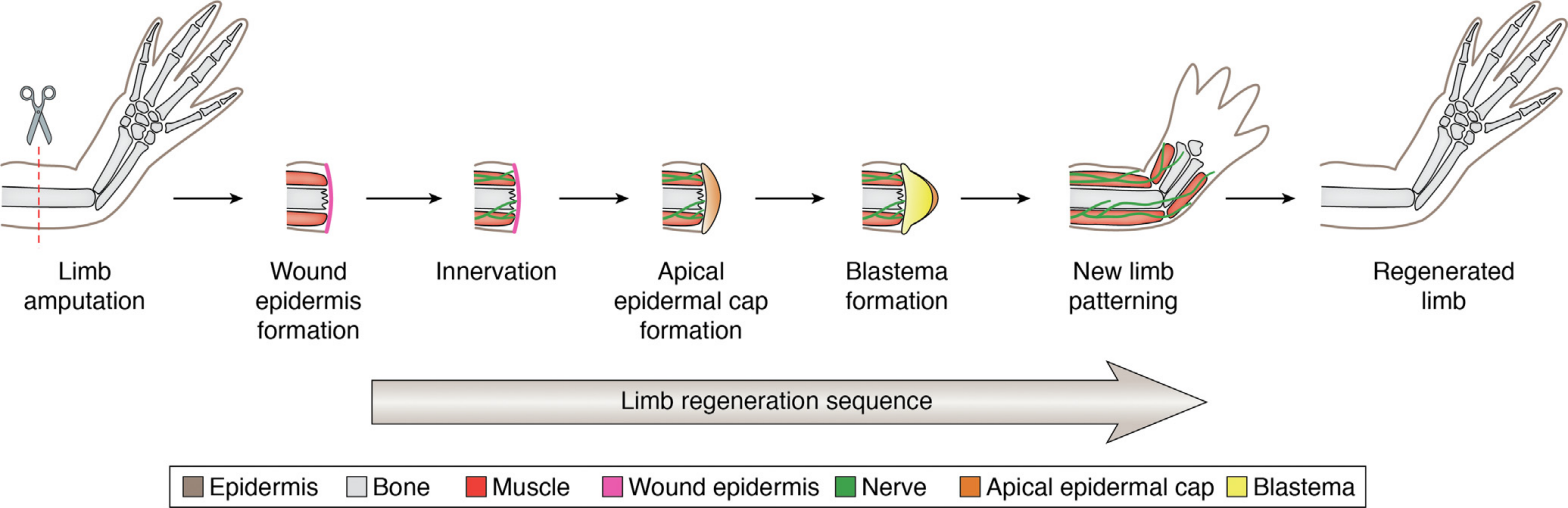
**Key stages of salamander limb regeneration.** Upon amputation, the epidermal cells migrate over the amputated site forming wound epidermis. Within days, wound epidermis becomes innervated which then becomes the apical epidermal cap (AEC). Blastema-forming cells are attracted to the AEC resulting a blastema formation which will continue to undergo proliferation and differentiation until a newly regenerated limb is formed. Figure adapted from McCusker *et al*. 2015 (147).

During regeneration, considerable changes take place, including stem and progenitor cell differentiation into multiple tissue types and reactivation of genes originally expressed during development (27, 32). Such processes are associated with signaling pathways that direct epigenetic reprogramming of the chromatin state through histone modification to alter the transcriptional landscape (33, 34). As genomic DNA is wrapped around histones, numerous different posttranslational modifications of histone tails occur regulating opening or closing of transcriptional regions during regeneration. These modifications include acetylation, methylation, phosphorylation, ubiquitination, and sumoylation (35). In addition, DNA methylation/demethylation is important as it is involved in epigenetic processes including gene expression, RNA splicing, and imprinting (36, 37, 38, 39, 40, 41). While extensive research has now focused on defining the repertoire and abundance of mRNAs expressed during limb regeneration, very little is known about the involvement of epigenetics in blastema formation. In this review, we will focus on currently known molecular and epigenetic regulation behind successful blastema formation.

## Wound healing in response to injury

Limb regeneration in salamanders is initiated in response to injury by forming the wound epidermis. Stress signals and inflammation (42, 43, 44) are upregulated upon trauma through multiple mechanisms which have not been fully elucidated. Following amputation, thrombin catalyzes the formation of fibrin clot to protect the wound tissue as well as provide temporary adhesive substrate for the epidermal cell migration (45, 46). The wound is usually closed 2 to 6 h post-amputation with migration of nonmitotic epidermal basal cells through the clot forming a wound epidermis (25). These migrating cells are primarily comprised of a single layer of keratinocytes (47) and are supplied from proximal mitotic epidermal cells (25, 48). The wound epidermis is distinguished by the expression of antigens WE3 and WE6 (49, 50) which are not expressed in uninjured epidermis, although the molecular identity of these antigens has not yet been determined. Similarly, keratin 5 (KRT5) and KRT17 is expressed in the basal layer during wound healing process and remains until late bud-blastema stage (51). Several studies have cataloged global gene expression in wound epidermis (52, 53, 54) and some have used gene expression in single cells to define cellular subtypes (16).

The importance of wound epidermis requirement for blastema formation has been demonstrated by suturing the skin flap over the amputated surface (55), repetitive removal of wound epidermis (56), or insertion of amputated stump into the body cavity (57), all of which lead to a block in outward blastema formation and, thus, regenerative failure. Scar tissue can also form when the formation of wound epidermis is experimentally blocked, which may further contribute to regenerative failure in salamander limbs (58, 59, 60). Repetitive limb removal has been shown to impair limb regeneration and to simultaneously promote a scar-like tissue composition, with accompanying changes to gene expression—reminiscent of scarring in mammalian contexts (61). These data imply the importance of scar-free healing promoted by the wound epidermis as a basis for blastema formation and regeneration. In mammals, wound healing typically results in scar tissue formation, with some exceptions: for example, scar-free wound healing has been observed in human fetal skin prior to the third trimester (62); the human oral cavity typically heals scar-free (although pathological complications can lead to scars (63)); the African spiny mouse has an incredible ability to fully restore its skin, hair follicles, sweat glands, dermis after large skin loss (64). Scar formation is a result of accumulation of collagenous fibers at wounded site (65), which is associated with transforming growth factor-beta (TGF-β) signaling affecting extracellular matrix organization (66). TGF-β has been identified as an important regulator of wound healing as it induces proliferation of skin fibroblasts and promotes migration of fibroblasts and keratinocytes (67, 68). In *Xenopus laevis* froglets, wounding triggers high expression of TGF-β, which results in scar-less wound healing (69). However, its expression is decreased in adults, resulting a scar-like tissue formation (69).

In axolotl salamanders, recent studies showed that important factors for wound epidermis formation without scarring are controlled by epigenetic regulation involving epithelial-to-mesenchymal transition (EMT)—a process in which the epithelial cells gain a motile mesenchymal phenotype by losing the junctions and polarity (70, 71). Pharmacological inhibition of both canonical and noncanonical TGF-β signaling showed significant decrease in EMT marker expression and a reduction in the rate of keratinocyte migration during wound closure (72). This study suggests that canonical and noncanonical TGF-β signaling regulates wound closure through epigenetic modification directing keratinocytes to migrate by undergoing EMT to lose their polarity and anchorage (70, 71). In addition, SALL4, a transcription factor which is not detectably expressed in uninjured tissue, has been identified to play a role in scar-free wound healing in axolotls. SALL4 expression is upregulated in cells localized to the wounded area of the epidermal, dermal, and muscle regions (73), where it regulates collagen transcription. In *Xenopus* tadpoles, SALL4 was shown to be highly expressed in the blastema during hindlimb regeneration (74). It has been suggested that SALL4 keeps the cells in an undifferentiated state in the blastema in both axolotls and *Xenopus* (73, 74). Experimental downregulation of SALL4 resulted in excessive collagen deposition at the wound site (73), a hallmark of scar formation (65). Interestingly, SALL4 has been previously shown to interact with transcription factors OCT4, NANOG, and SOX2 (75, 76, 77), which function in cellular reprogramming through altering epigenetic landscape (78, 79). Currently, the cooperative role of TGF-β and SALL4, if any, in wound healing is unclear. However, these studies underscore the importance of epigenetic modification in proregenerative wound healing.

## Blastema formation

### Innervation is required for AEC formation

Within 2 to 3 days post-amputation, wound epidermis becomes innervated as cells accumulate and the apex thickens (80, 81). In response to nerve signals, wound epidermis matures and is then referred to as the apical epidermal cap (AEC). The AEC undergoes several additional changes, including stratification, prior to—or in parallel with—blastema formation (82).

Innervation of the wound epithelium is a critical event directing regeneration as denervated limbs do not regenerate (83) but instead heal with a densely packed scar-like layer of cells (22, 23). The importance of innervation in regeneration was also observed in the accessory limb model (ALM). This model system is a proxy for limb regeneration insofar as it seeks to experimentally define what tissues or molecular signaling components are sufficient for growth of new limbs (84). In this method, skin from the posterior region of the limb is grafted to the anterior region of the contralateral limb, and brachial nerve is surgically deviated to the site of this experimentally prepared anterior skin wound. This operation results in the formation of an ectopic blastema equivalent to an amputation-induced blastema, which, in the best cases, goes on to develop into a new limb (84). However, if the nerve is not deviated toward the skin graft, the ectopic blastema is not formed and instead scar-free wound healing occurs (84). Ectopic limb formation is therefore made possible due to the positional disparity between grafted cells and those at the wounded site, in collaboration with nerves. This interaction stimulates the intercalation of an amputated limb field, thus generating an ectopic limb.

Using the ALM system, the importance of innervation was supported by investigating the role of *buttonhead*-like zinc-finger transcription factor *SP9* in limb regeneration. *SP9* is expressed in the apical ectoderm of developing limb buds of the larva and regulates the outgrowth of the developing limb (85). An ALM assay demonstrated that *SP9* expression is dependent on innervation as *SP9* is reexpressed after amputation in the basal keratinocytes of the wound epidermis and remains continuously expressed during limb regeneration (86). However, another study discovered that ALM with nondeviated nerves show ectopic limb generation when treated with FGFs and BMPs – FGF2, FGF8, and BMP2 or BMP7 promoted the most robust rates of limb formation (87, 88). FGF2, BMP2, and BMP7 are neurotrophic factors (89), and FGF8 is expressed in both the basal layer of the AEC (87, 89) and the dorsal root ganglion (where sensory nerve cell bodies reside) (90). This study suggests that FGF2, FGF8, and BMP2 or BMP7 may perhaps be sufficient to generate ectopic limb in the absence of innervation.

### Epigenetic regulation during AEC formation

**Figure 2:**
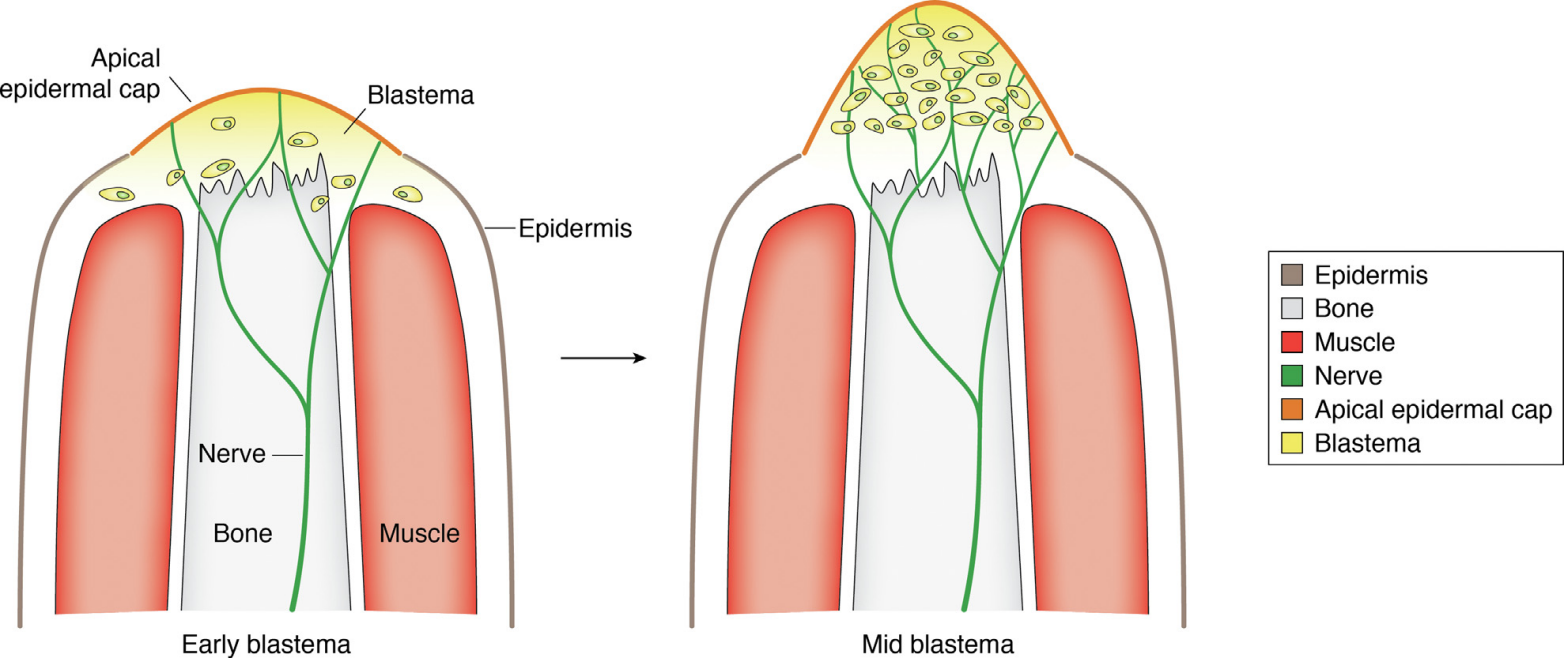
**Blastema formation with the migration of blastema-forming cells.** Newly activated blastema-forming cells migrate towards the stump forming the blastema.

**Figure 3:**
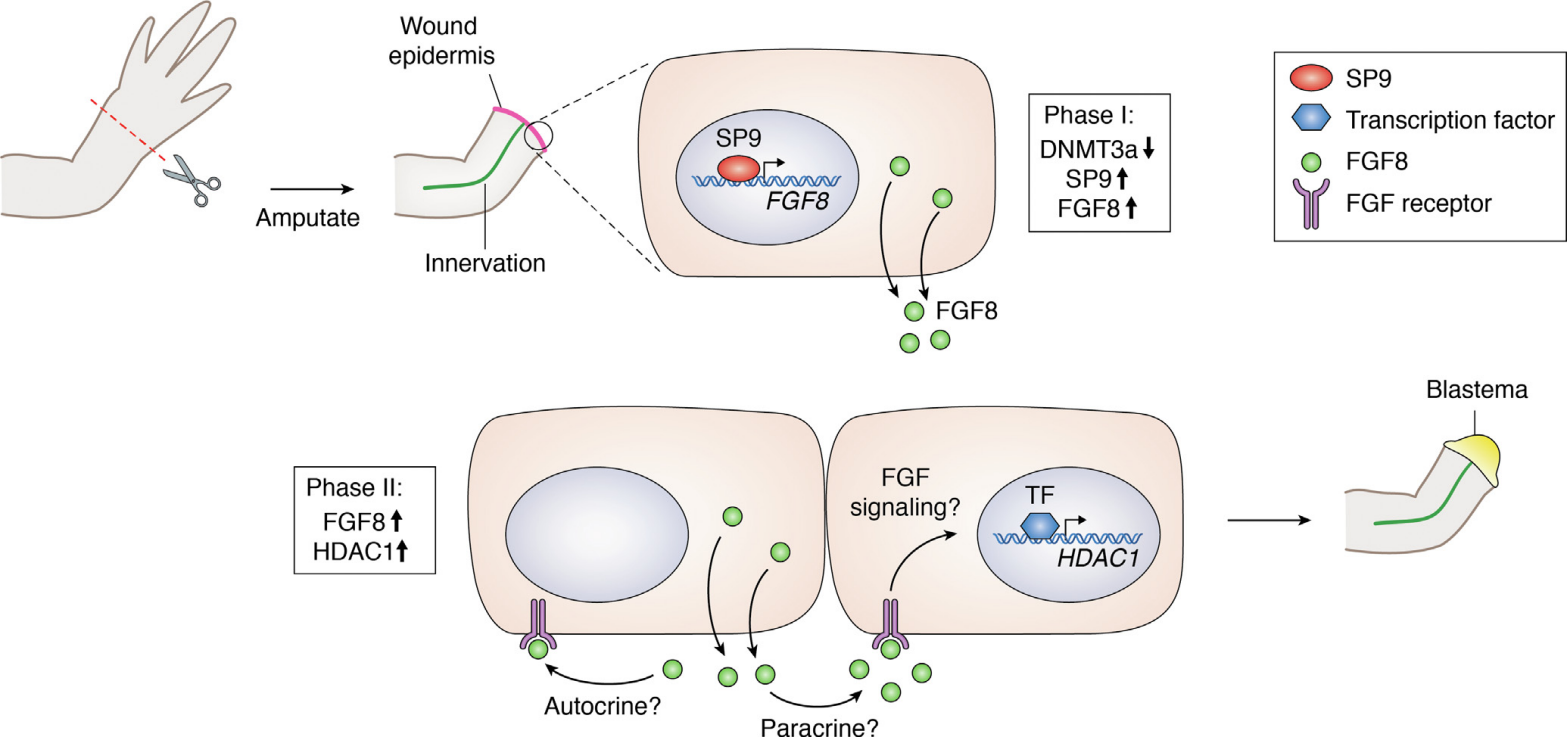
**Proposed epigenetic regulation of blastema formation after wound healing.** (Phase I, *top*) Innervation downregulates DNMT3a in the wound epidermis which then leads to initiation of *FGF8* transcription by transcription factor SP9. FGF8 is then released to extracellular matrix. (Phase II, *bottom*) FGF8 present in the extracellular matrix initiates cell signaling through autocrine or paracrine signaling to induce HDAC1 contributing to blastema formation. DNMT3a, DNA methyltransferase 3a; FGF, fibroblast growth factor; HDAC, histone deacetylase.

Nerve signaling promotes wound epidermis transition to AEC, and denervation or lack of wound epidermis results in failure to form the blastema (55, 57, 86). During the process of AEC formation, subjacent tissues to the wound epidermis including those harboring fibroblasts, muscle, and skeletal cells undergo activation, reenter the cell cycle, commence proliferation, and, in cases of those destined to become blastema cells, migrate to the tip of the stump (91) (Fig. 2). Upon injury, *SP9* expression is reinitiated by reopening of the gene’s regulatory region, allowing access to regulatory factors to initiate transcription (92). This event demonstrates that keratinocytes undergo nerve-dependent dedifferentiation into a limb bud–like state through epigenetic modification and mimicking the limb development stage. In the ALM system, innervation was shown to downregulate DNA methyltransferase 3a (DNMT3a) expression in the wound epidermis and subsequently enhance *SP9* expression (92). As DNMT3a can perform *de novo* methylation of CpG islands (93), decrease in DNMT3a expression during innervation could be required for the wound epidermis to differentiate and acquire the signaling properties of the AEC, perhaps by preventing methylation of SP9 regulatory regions (86). In contrast, histone deacetylase 1 (HDAC1), which functions to repress transcription by assembling heterochromatin through histone deacetylation (94), was upregulated in the wound epithelium (95, 96). Conversely, denervation prior to amputation or HDAC1 inhibition impeded regeneration (95, 96), in an agreement with previous studies where HDAC inhibition hampered regeneration of axolotl larvae tail and *Xenopus* tadpole tail and limbs (97, 98, 99). This study further reported that treatment with neurotrophic factors FGF2, FGF8, and BMP7, introduced earlier (87), resulted in a partial rescue in HDAC1 expression and limb regeneration. Interestingly, *FGF8*, which is expressed in both dorsal root ganglion and AEC basal cells (87, 89, 90), was shown to be regulated by SP9 during embryonic limb development (85). Taken together, it is possible that, through concerted epigenetic regulation of gene expression, neurotrophic factors may repress DNMT3a to subsequently upregulate *SP9* and *FGF8* expression and thus activate HDAC1 for regeneration (Fig. 3). However, this hypothesis needs additional experimentation to be fully tested.

## Blastema formation and growth

### Dedifferentiated blastema cells exhibit lineage restriction

**Figure 4:**
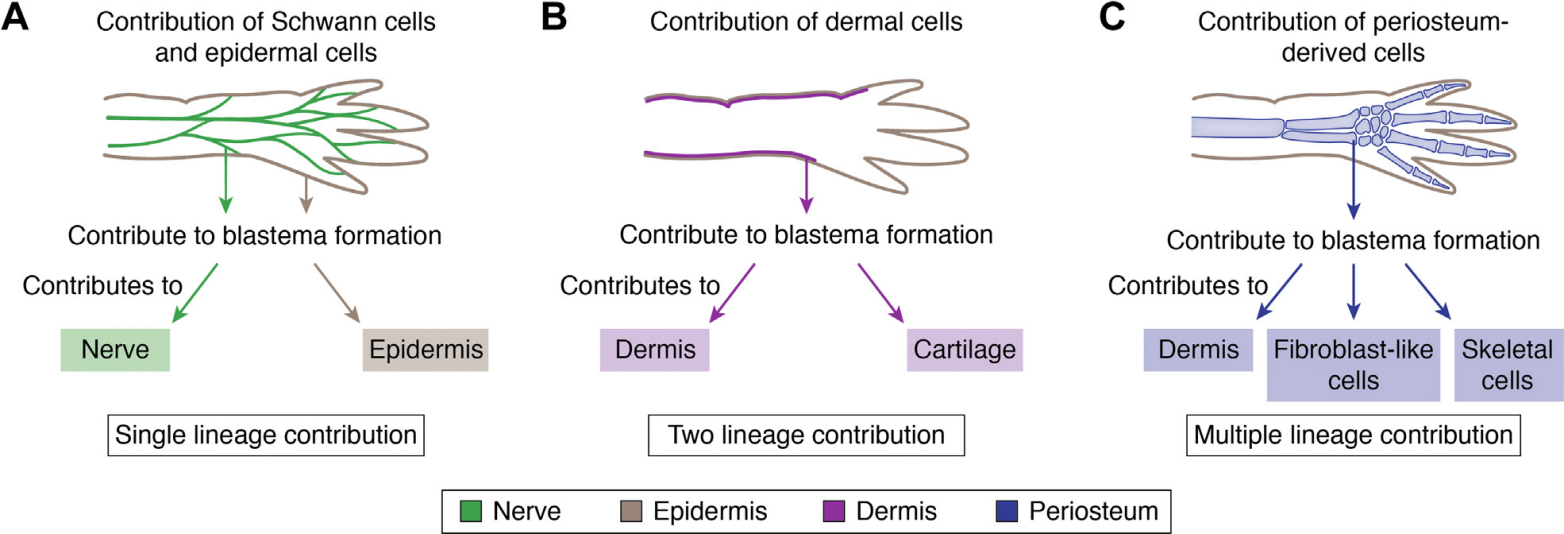
**Contribution map of various cells during limb regeneration.***A*, Schwann cells and epidermal cells both contribute to blastema formation. However, Schwann cells and epidermal cells are lineage restricted contributing to itself. *B*, dermal cells contribute to blastema and then behaves as bipotent progenitor cell giving rise to dermal and cartilage. *C*, periosteum-derived cells are multipotent which contributes to blastema then regenerates dermal, fibroblast-like, and skeletal cells (cartilage, bone, periosteum).

Following AEC formation, blastema progenitor cells accumulate to form the early blastema. Prior to blastema formation, resident cells at the wounded site reenter the cell cycle (100, 101), leading to proliferation. To examine the mechanistic property of cycling cells (4N), the transcriptional profiles of cycling cells were compared to that of the wound epidermis and nondividing cells (2N). Surprisingly, pathway analysis revealed that several growth factor signaling pathways are suppressed in 4N cell population, while HIPPO, WNT, and TGF-β signaling, which are important in wound epidermis and blastema formation, were upregulated (72, 102, 103). To examine which tissues contribute to blastema formation, transplant experiments have been performed. Early transplantation experiments leveraged experimentally created triploid axolotls, with tissues grafted into typical diploid hosts (26, 104). More recent chimeras have been generated using transgenic axolotls with ubiquitous GFP expression, often taking advantage of existing embryonic fate maps in the experimental design (13, 105, 106, 107). For example, transplanting GFP-labeled Schwann cells and epidermal cells showed contribution to blastema formation, and grafts indicated these cell types produce daughter cells of similar cell types in the regenerate limb (108) (Fig. 4*A*). Interestingly, dermal cells initially contributed to blastema but later differentiated into dermal and cartilage, demonstrating acquisition of bipotency (26, 108) (Fig. 4*B*).

More refined lineage tracing is enabled by genetic labeling methods, and these are now being applied in salamander systems. Muscle cells have been documented to have different activities upon amputation depending on type and life stage of salamander. While both larval and adult newts have muscle satellite cells, which serve as stem cells for muscle regeneration in many other systems (109, 110, 111), only larval newts utilize satellite cells for limb regeneration, whereas mononucleated myofibers are dedifferentiated to serve as muscle progenitors in adult newts (112, 113). It is not clear why a dedifferentiation strategy is used exclusively in adults. Possible explanations might include compartmentalization of function, a scenario in which satellite cells are used for muscle maintenance whereas dedifferentiation is used for regeneration upon injury.

Unlike tissues such as skin and muscle, which show no evidence of transdifferentiation, periosteum-derived cells contribute to the blastema formation, but their ultimate contribution to the regenerate limb appears in descendent cells populating numerous tissues including skeletal (cartilage, bone, periosteum), dermal, and fibroblast-like tissue (114) (Fig. 4*C*). Transplant studies using cartilage demonstrated that cartilage can contribute to cartilage, tendons, and perichondrium (108). However, whether the contribution of cartilage is attributable to inclusion of perichondrium or not is still unclear.

From these studies, it could be concluded that the blastema is a heterogenous tissue consisting of multipotent and lineage-restricted progenitor cells, some of which arise *via* stem cell activation and some of which may arise *via* dedifferentiation. Blastema cells may also be derived from the activation of uncharacterized cells with stem cell-like properties. In cases of either stem cell activation or dedifferentiation, it remains unclear whether progenitor cells randomly undergo activation or dedifferentiation to form blastema or whether there are specific cells from each lineage that are reserved for use as blastemal progenitors. Each of these possible scenarios could theoretically be influenced by epigenetic mechanisms that await discovery.

### Maintaining positional memory is essential for axolotl limb regeneration

**Figure 5:**
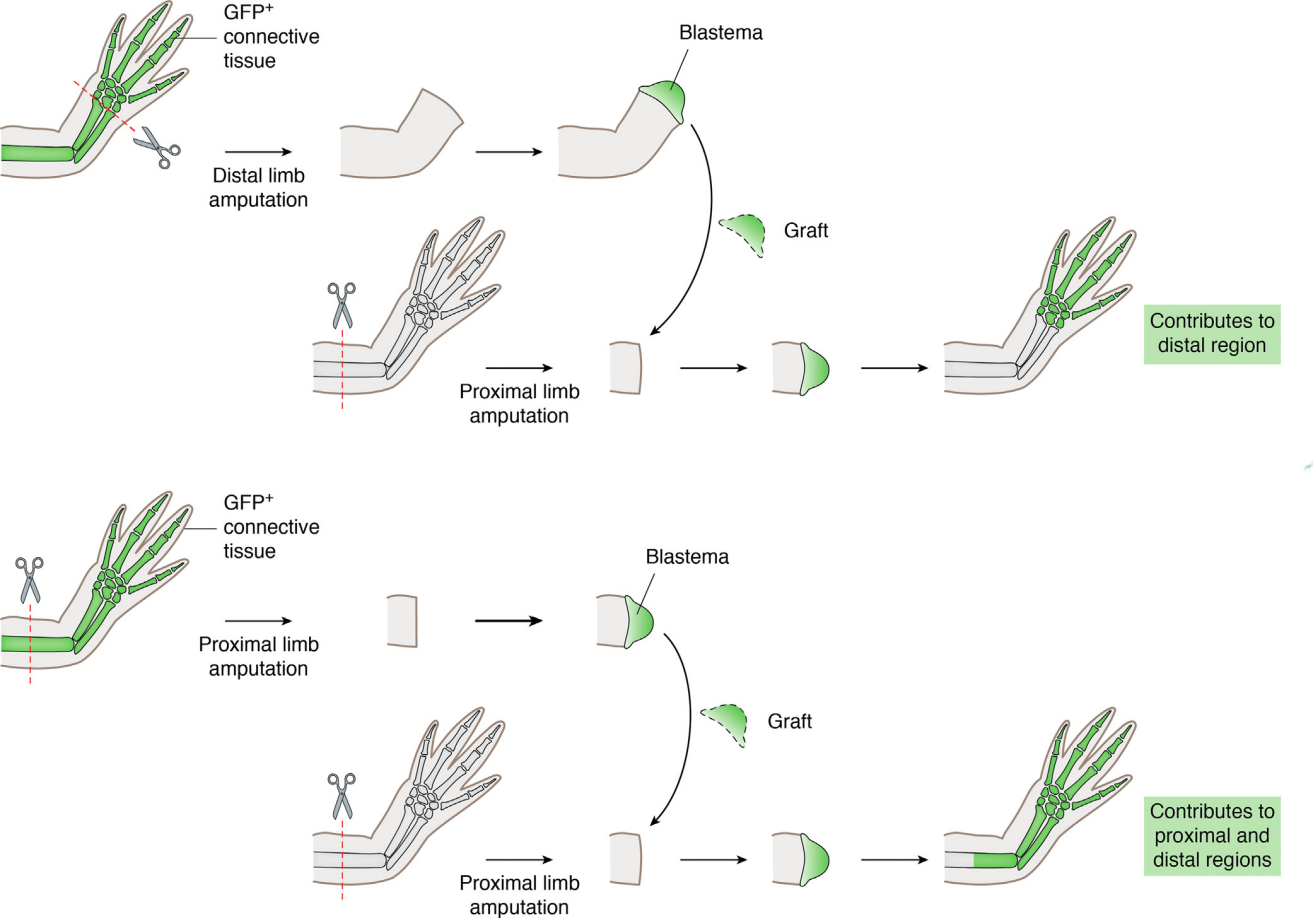
**Positional memory is maintained in the cell.** Cartoon showing that positional memory is maintained in each cell. (*Top*) Transplanting GFP^+^ distal blastema to proximal amputated region shows that GFP^+^ blastema cell contribution is limited to host hand. (*Bottom*) GFP^+^ proximal blastema transplanted to proximal amputated region shows that GFP^+^ blastema cells contribute to the entire host limb.

In addition to lineage restriction, dedifferentiated axolotl blastema cells possess positional memory, a molecular system for maintaining the position of origin in relation to neighboring cells (115) (Fig. 5). Positional memory is restricted to fibroblast-derived dedifferentiated cells, including cartilage cells, which form the blastema (108, 116, 117, 118). These cells proliferate and differentiate by the rule of distal transformation, forming only the lost structures (108, 118). Positional memory is apparent after individually transplanting undifferentiated blastemas from wrist, elbow, and mid-upper arm levels of the forelimb to the blastema-stump junction of hindlimbs. The results showed that wrist, elbow, and mid-upper arm level blastemas were directed correctly to the corresponding hindlimb region—ankle, knee, and femur, respectively (119). These outcomes were altered by retinoic acid treatment, which causes proximalization of transplanted blastemas (119), similar to its effects in bullfrog and axolotl limb regeneration following simple amputation (120, 121, 122). It is suggested that retinoic acid treatment causes proximalization through upregulation of genes that are expressed in proximal regions (123). Those include stylopod-specific transcription factors MEIS1/2 (124, 125, 126) and PBX1 (127). In addition, genes that are expressed in the distal region and required for distal identity including HOXA13 (108, 127), LHX9 (128), and SPRY1 (129) were downregulated (123). A more recent study further investigated experimental proximalization (130) using *SHOX2*, which had previously been discovered to serve as a marker gene whose expression is enriched in blastemas derived from proximal amputations *versus* those derived from distal amputations (15). Upon grafting a full-thickness wrist skin on the amputated stylopod (upper arm or thigh), in which the host skin was stripped prior to amputation, *SHOX2* expression was detected in the blastema despite the fact that grafted tissue was of distal origin (130). These studies indicate a possible role for epigenetic regulation in maintaining positional memory of cells during limb regeneration, but additional work is necessary to support this prediction.

Not all cells are thought to have positional memory, and our current knowledge about the kind of cellular programming needed to maintain positional memory is lacking. As recently reported, anteroposterior and dorsoventral skin grafting seems to show minimal effect in positional memory. During limb regeneration, *SHH* is expressed in the posterior region of the blastema. However, only a few cells from the blastema were able to express *SHH* when GFP-labeled anterior skin was grafted to the posterior side prior to amputation (130). On the contrary, *SHH* expression remained in the anterior side of the blastema upon grafting GFP-labeled posterior skin to anterior side (130). Similar stability in positional memory was also seen after transplanting a GFP-labeled wrist blastema to an amputated upper arm of non-GFP axolotl. In this study, only the hand displayed GFP expression (118). When GFP-labeled cartilage from the fingertip is transplanted to the upper arm, GFP-labeled blastema cells showed contribution only to the hand (108). In contrast, the muscle- and Schwann cell–derived blastema lacks positional memory as their regenerative pattern initiated immediately distal from the recipient blastema (108). From these data, it is possible that positional memory of each tissue type is maintained differently, and it is also possible that each tissue shows tissue-specific cellular behavior in limb regeneration.

During limb development, the stylopod (upper arm or thigh), zeugopod (lower arm or leg), and autopod (hand or foot) are specified by MEIS, HOXA11, and HOXA13 expression (131). When upper arm blastemas or wrist blastemas are transplanted and stained for MEIS and HOXA3, a great number of upper arm–derived blastema cells are MEIS^+^ at the proximal end, whereas wrist-derived blastema cells show restricted expression of HOXA13 at the distal end (108). When Schwann cell–derived blastema cells are stained, both MEIS and HOXA13 expression was not observed, indicating that Schwann cells do not have position-specific identity.

### Positional memory of blastema cells may be retained by epigenetic memory

Among the blastema cells, not all cells must theoretically possess both progenitor cell properties and positional memory. As each blastema cell theoretically may retain a different positional memory, distinct epigenetic states may be required for maintenance. Despite such possible intricate regulation, not much is known about the epigenetic landscape of the blastema and its modification during different stages of limb regeneration.

In *X. laevis*, limb regeneration occurs in a similar manner as axolotls by forming a blastema which is thought to obtain the limb patterning properties from the stump cells and is retained throughout regeneration (132). To test whether the patterning information is retained as epigenetic memory, the histone marks including histone H3 at lysine 4 trimethylation (H3K4me3) and histone H3 at lysine 27 trimethylation (H3K27me3) were examined in developing limb bud and regenerative blastema (133). When histone modification profiles of developing limb buds and regenerating blastemas were compared through a genome-wide comprehensive analysis, both limb bud and blastema cells showed similar histone marks. These results support that histone modifications are maintained as epigenetic memory (133).

Although axolotls also show that the patterning mechanism in development and regeneration are largely similar as in *Xenopus* (31), conducting this experiment in axolotls is important to determine if the outcome is similar. Future comparative studies examining of epigenetic landscape that occur in cells with different positional memories during regeneration and development will provide further insights into the epigenetic mechanisms underlying limb regeneration.

## Conclusion and future perspective

Limb regeneration is a dynamic cellular process which is dependent on blastema formation followed by subsequent proliferation and differentiation. Once the blastema is created, the regeneration process mimics the limb development program from several perspectives, albeit on a different scale. The gene expression changes underlying these events imply that epigenetic modifications and relationships may be key upstream controls. As epigenetics changes play a large role in biological process at the chromatin reorganization and transcription level, histone posttranslational modifications or DNA modifications lead to “open” or “closed” chromatin, leading to changes in gene expression (35). As previously discussed, current knowledge is limited to the role of DNMT3a and HDAC1, which has been investigated in ALM (92) and during tail/limb regeneration (95, 96, 97), respectively. Unfortunately, compared to other organs and tissues from other organisms, epigenetic mechanisms are not still well understood and the science addressing these issues in salamander limb regeneration is still in its infancy. However, a study leveraging ATAC-seq, which quantifies chromatin accessibility of DNA based on transposase insertion *in vitro*, was recently performed to profile the chromatin accessibility dynamics of the blastema and redifferentiated cells (134). As this study provides temporal enrichment of transcription factors, further complementary studies profiling histone modifications to understand important aspects of regeneration including blastema formation, positional memory, and cellular reprogramming may arise based on this data. Therefore, elucidating epigenetic controls of limb regeneration promises to inform regenerative biology and, ultimately, regenerative medicine.

Axolotls show remarkable ability to regenerate different body parts including limbs, tail, upper and lower jaw, lens, and many others (3, 135, 136, 137). Perhaps due to their multiple regenerative abilities, axolotl cells show plasticity during regeneration and can even be coaxed to generate supernumerary limbs, as shown with ALM. Taking advantage of these plastic cellular properties, experimentally modulating the epigenetic landscape of stump cells in nonregenerative contexts might enable regeneration. Possible starting point for such explorations are contexts in which axolotl regeneration is blocked, which includes repeated amputation (61) or full-thickness skin flap suturing across the amputation plane (55). Insights from these kinds of approaches might then be applied to the stumps of otherwise non-regenerative extant animals, such as mammals.

In mammals, digit tip is a comparably well-studied model for regeneration and shares similar regenerative phases with axolotl limb regeneration including inflammation, histolysis, wound closure, blastema formation, and differentiation to restore amputated part (138, 139). Unlike axolotls, which have a pool of both multipotent and lineage-specific cells in the blastema, mouse digit tip regeneration occurs through lineage-restricted progenitor cells (140, 141). In addition, digit tip regeneration proceeds without innervation (142) implying that nerve-mediated signals are not necessary for the initial step of regeneration. However, it is currently unclear how the pool of lineage-restricted mouse stem and progenitor cells could be induced to have similar regenerative potential to salamanders. Perhaps given the restricted regenerative capacity after mouse digit tip amputation, reprogramming digit tip cells into pluripotent stem cells akin to limb bud cells might enable regeneration following more proximal amputations (143, 144, 145, 146). Another, non mutually-exclusive possibility is that mammalian cells can be tweaked using epigenetic approaches to express genes necessary for salamander limb regeneration. These ideas are exciting to consider, and research investigating the natural cellular reprogramming that occurs during limb regeneration, leveraging new tools to probe genomics and epigenomics, is likely to explode in coming years and decades.

## Conflict of interest

The authors declare that they have no conflicts of interest with the contents of this article.

## References

[1] Dalyell J.G. (1814).

[2] Morgan T.H. (1898). Experimental studies of the regeneration of Planaria maculata. Roux's Arch. Dev. Biol..

[3] Young H.E., Bailey C.F., Dalley B.K. (1983). Gross morphological analysis of limb regeneration in postmetamorphic adult Ambystoma. Anat. Rec..

[4] Morgan T.H. (1900). Regeneration in teleosts. Archiv für Mikroskopische Anatomie.

[5] Michalopoulos G.K. (2007). Liver regeneration. J. Cell Physiol..

[6] Philip S.J., Kumar R.J., Menon K.V. (2005). Morphological study of rib regeneration following costectomy in adolescent idiopathic scoliosis. Eur. Spine J..

[7] Illingworth C.M. (1974). Trapped fingers and amputated finger tips in children. J. Pediatr. Surg..

[8] Ipsen T., Frandsen P.A., Barfred T. (1987). Conservative treatment of fingertip injuries. Injury.

[9] Jafari P., Muller C., Grognuz A., Applegate L.A., Raffoul W., di Summa P.G. (2017). First insights into human fingertip regeneration by Echo-Doppler imaging and wound microenvironment assessment. Int. J. Mol. Sci..

[10] Shieh S.J., Cheng T.C. (2015). Regeneration and repair of human digits and limbs: fact and fiction. Regeneration (Oxf).

[11] Schloissnig S., Kawaguchi A., Nowoshilow S., Falcon F., Otsuki L., Tardivo P. (2021). The giant axolotl genome uncovers the evolution, scaling, and transcriptional control of complex gene loci. Proc. Natl. Acad. Sci. U. S. A..

[12] Nowoshilow S., Schloissnig S., Fei J.F., Dahl A., Pang A.W.C., Pippel M. (2018). The axolotl genome and the evolution of key tissue formation regulators. Nature.

[13] Sobkow L., Epperlein H.H., Herklotz S., Straube W.L., Tanaka E.M. (2006). A germline GFP transgenic axolotl and its use to track cell fate: dual origin of the fin mesenchyme during development and the fate of blood cells during regeneration. Dev. Biol..

[14] Whited J.L., Tsai S.L., Beier K.T., White J.N., Piekarski N., Hanken J. (2013). Pseudotyped retroviruses for infecting axolotl *in vivo* and *in vitro*. Development.

[15] Bryant D.M., Johnson K., DiTommaso T., Tickle T., Couger M.B., Payzin-Dogru D. (2017). A tissue-mapped axolotl *de novo* transcriptome enables identification of limb regeneration factors. Cell Rep..

[16] Leigh N.D., Dunlap G.S., Johnson K., Mariano R., Oshiro R., Wong A.Y. (2018). Transcriptomic landscape of the blastema niche in regenerating adult axolotl limbs at single-cell resolution. Nat. Commun..

[17] Stewart R., Rascón C.A., Tian S., Nie J., Barry C., Chu L.F. (2013). Comparative RNA-seq analysis in the unsequenced axolotl: the oncogene burst highlights early gene expression in the blastema. PLoS Comput. Biol..

[18] Fei J.F., Schuez M., Knapp D., Taniguchi Y., Drechsel D.N., Tanaka E.M. (2017). Efficient gene knockin in axolotl and its use to test the role of satellite cells in limb regeneration. Proc. Natl. Acad. Sci. U. S. A..

[19] Voss S.R., Palumbo A., Nagarajan R., Gardiner D.M., Muneoka K., Stromberg A.J. (2015). Gene expression during the first 28 days of axolotl limb regeneration I: experimental design and global analysis of gene expression. Regeneration (Oxf).

[20] Keinath M.C., Timoshevskiy V.A., Timoshevskaya N.Y., Tsonis P.A., Voss S.R., Smith J.J. (2015). Initial characterization of the large genome of the salamander Ambystoma mexicanum using shotgun and laser capture chromosome sequencing. Sci. Rep..

[21] Monaghan J.R., Epp L.G., Putta S., Page R.B., Walker J.A., Beachy C.K. (2009). Microarray and cDNA sequence analysis of transcription during nerve-dependent limb regeneration. BMC Biol..

[22] Salley J.D., Tassava R.A. (1981). Responses of denervated adult newt limb stumps to reinnervation and reinjury. J. Exp. Zool.

[23] Tassava R.A., Loyd R.M. (1977). Injury requirement for initiation of regeneration of newt limbs which have whole skin grafts. Nature.

[24] TJ T. (1823). On the process of reproduction of the members of aquatic salamander. Q. J. Sci. Literat. Arts.

[25] Hay E.D., Fischman D.A. (1961). Origin of the blastema in regenerating limbs of the newt Triturus viridescens. An autoradiographic study using tritiated thymidine to follow cell proliferation and migration. Dev. Biol..

[26] Gardiner D.M., Muneoka K., Bryant S.V. (1986). The migration of dermal cells during blastema formation in axolotls. Dev. Biol..

[27] Bryant S.V., Endo T., Gardiner D.M. (2002). Vertebrate limb regeneration and the origin of limb stem cells. Int. J. Dev. Biol..

[28] Gardiner D.M., Endo T., Bryant S.V. (2002). The molecular basis of amphibian limb regeneration: integrating the old with the new. Semin. Cell Dev. Biol..

[29] Rose S.M. (1962). Regeneration.

[30] Maden M. (1980). Intercalary regeneration in the amphibian limb and the rule of distal transformation. J. Embryol. Exp. Morphol..

[31] Muneoka K., Bryant S.V. (1982). Evidence that patterning mechanisms in developing and regenerating limbs are the same. Nature.

[32] Géraudie J., Ferretti P. (1998). Gene expression during amphibian limb regeneration. Int. Rev. Cytol..

[33] Bonasio R., Tu S., Reinberg D. (2010). Molecular signals of epigenetic states. Science.

[34] Arzate-Mejía R.G., Valle-García D., Recillas-Targa F. (2011). Signaling epigenetics: novel insights on cell signaling and epigenetic regulation. IUBMB Life.

[35] Kouzarides T. (2007). Chromatin modifications and their function. Cell.

[36] Wu H., D'Alessio A.C., Ito S., Wang Z., Cui K., Zhao K. (2011). Genome-wide analysis of 5-hydroxymethylcytosine distribution reveals its dual function in transcriptional regulation in mouse embryonic stem cells. Genes Dev..

[37] Zhu W.G., Srinivasan K., Dai Z., Duan W., Druhan L.J., Ding H. (2003). Methylation of adjacent CpG sites affects Sp1/Sp3 binding and activity in the p21(Cip1) promoter. Mol. Cell Biol..

[38] Laurent L., Wong E., Li G., Huynh T., Tsirigos A., Ong C.T. (2010). Dynamic changes in the human methylome during differentiation. Genome Res..

[39] Anastasiadou C., Malousi A., Maglaveras N., Kouidou S. (2011). Human epigenome data reveal increased CpG methylation in alternatively spliced sites and putative exonic splicing enhancers. DNA Cell Biol..

[40] Guo J.U., Su Y., Zhong C., Ming G.L., Song H. (2011). Hydroxylation of 5-methylcytosine by TET1 promotes active DNA demethylation in the adult brain. Cell.

[41] Hackett J.A., Sengupta R., Zylicz J.J., Murakami K., Lee C., Down T.A. (2013). Germline DNA demethylation dynamics and imprint erasure through 5-hydroxymethylcytosine. Science.

[42] Godwin J.W., Pinto A.R., Rosenthal N.A. (2013). Macrophages are required for adult salamander limb regeneration. Proc. Natl. Acad. Sci. U. S. A..

[43] Lewis J.L., Sullivan A.M. (2020). Salamander stress and duress: the relationship between CORT, autotomy and regeneration, and exploratory behaviour. Zoology (Jena).

[44] Love N.R., Chen Y., Ishibashi S., Kritsiligkou P., Lea R., Koh Y. (2013). Amputation-induced reactive oxygen species are required for successful Xenopus tadpole tail regeneration. Nat. Cell Biol..

[45] Repesh L.A., Furcht L.T., Smith D. (1981). Immunocytochemical localization of fibronectin in limb tissues of the adult newt, Notophthalmus viridescens. J. Histochem. Cytochem..

[46] Donaldson D.J., Mahan J.T., Hasty D.L., McCarthy J.B., Furcht L.T. (1985). Location of a fibronectin domain involved in newt epidermal cell migration. J. Cell Biol..

[47] Repesh L.A., Oberpriller J.C. (1978). Scanning electron microscopy of epidermal cell migration in wound healing during limb regeneration in the adult newt, Notophthalmus viridescens. Am. J. Anat..

[48] Lash J.W. (1955). Studies on wound closure in urodeles. J. Exp. Zoolog..

[49] Castilla M., Tassava R.A. (1992). Extraction of the WE3 antigen and comparison of reactivities of mAbs WE3 and WE4 in adult newt regenerate epithelium and body tissues. Monogr. Dev. Biol..

[50] Estrada C.M., Park C.D., Castilla M., Tassava R.A. (1993). Monoclonal antibody WE6 identifies an antigen that is up-regulated in the wound epithelium of newts and frogs. Prog. Clin. Biol. Res..

[51] Moriyasu M., Makanae A., Satoh A. (2012). Spatiotemporal regulation of keratin 5 and 17 in the axolotl limb. Dev. Dyn..

[52] Knapp D., Schulz H., Rascon C.A., Volkmer M., Scholz J., Nacu E. (2013). Comparative transcriptional profiling of the axolotl limb identifies a tripartite regeneration-specific gene program. PLoS One.

[53] Campbell L.J., Suárez-Castillo E.C., Ortiz-Zuazaga H., Knapp D., Tanaka E.M., Crews C.M. (2011). Gene expression profile of the regeneration epithelium during axolotl limb regeneration. Dev. Dyn..

[54] Monaghan J.R., Athippozhy A., Seifert A.W., Putta S., Stromberg A.J., Maden M. (2012). Gene expression patterns specific to the regenerating limb of the Mexican axolotl. Biol. Open.

[55] Mescher A.L. (1976). Effects on adult newt limb regeneration of partial and complete skin flaps over the amputation surface. J. Exp. Zool.

[56] Thornton C.S. (1957). The effect of apical cap removal on limb regeneration in Amblystoma larvae. J. Exp. Zool.

[57] Goss R.J. (1956). Regenerative inhibition following limb amputation and immediate insertion into the body cavity. Anat. Rec..

[58] Butler E.G., Schotté O.E. (1941). Histological alterations in denervated non-regenerating limbs of urodele larvae. J. Exp. Zoolog..

[59] Schotté O.E., Butler E.G. (1941). Morphological effects of denervation and amputation of limbs in urodele larvae. J. Exp. Zoolog..

[60] Singer M., Craven L. (1948). The growth and morphogenesis of the regenerating forelimb of adult Triturus following denervation at various stages of development. J. Exp. Zool.

[61] Bryant D.M., Sousounis K., Payzin-Dogru D., Bryant S., Sandoval A.G.W., Martinez Fernandez J. (2017). Identification of regenerative roadblocks *via* repeat deployment of limb regeneration in axolotls. NPJ Regen. Med..

[62] Longaker M.T., Whitby D.J., Adzick N.S., Crombleholme T.M., Langer J.C., Duncan B.W. (1990). Studies in fetal wound healing, VI. Second and early third trimester fetal wounds demonstrate rapid collagen deposition without scar formation. J. Pediatr. Surg..

[63] Turksen K. (2018).

[64] Seifert A.W., Kiama S.G., Seifert M.G., Goheen J.R., Palmer T.M., Maden M. (2012). Skin shedding and tissue regeneration in African spiny mice (Acomys). Nature.

[65] Ehrlich H.P., Krummel T.M. (1996). Regulation of wound healing from a connective tissue perspective. Wound Repair Regen..

[66] Roberts A.B., McCune B.K., Sporn M.B. (1992). TGF-beta: regulation of extracellular matrix. Kidney Int..

[67] Gailit J., Welch M.P., Clark R.A. (1994). TGF-beta 1 stimulates expression of keratinocyte integrins during re-epithelialization of cutaneous wounds. J. Invest. Dermatol..

[68] Lévesque M., Gatien S., Finnson K., Desmeules S., Villiard E., Pilote M. (2007). Transforming growth factor: beta signaling is essential for limb regeneration in axolotls. PLoS One.

[69] Bertolotti E., Malagoli D., Franchini A. (2013). Skin wound healing in different aged Xenopus laevis. J. Morphol..

[70] Skrypek N., Goossens S., De Smedt E., Vandamme N., Berx G. (2017). Epithelial-to-Mesenchymal transition: epigenetic reprogramming driving cellular plasticity. Trends Genet..

[71] Lamouille S., Xu J., Derynck R. (2014). Molecular mechanisms of epithelial-mesenchymal transition. Nat. Rev. Mol. Cell Biol..

[72] Sader F., Denis J.F., Laref H., Roy S. (2019). Epithelial to mesenchymal transition is mediated by both TGF-β canonical and non-canonical signaling during axolotl limb regeneration. Sci. Rep..

[73] Erickson J.R., Gearhart M.D., Honson D.D., Reid T.A., Gardner M.K., Moriarity B.S. (2016). A novel role for SALL4 during scar-free wound healing in axolotl. NPJ Regen. Med..

[74] Neff A.W., King M.W., Harty M.W., Nguyen T., Calley J., Smith R.C. (2005). Expression of Xenopus XlSALL4 during limb development and regeneration. Dev. Dyn..

[75] Forghanifard M.M., Ardalan Khales S., Javdani-Mallak A., Rad A., Farshchian M., Abbaszadegan M.R. (2014). Stemness state regulators SALL4 and SOX2 are involved in progression and invasiveness of esophageal squamous cell carcinoma. Med. Oncol..

[76] Wu Q., Chen X., Zhang J., Loh Y.H., Low T.Y., Zhang W. (2006). Sall4 interacts with Nanog and co-occupies Nanog genomic sites in embryonic stem cells. J. Biol. Chem..

[77] Tan M.H., Au K.F., Leong D.E., Foygel K., Wong W.H., Yao M.W. (2013). An Oct4-Sall4-Nanog network controls developmental progression in the pre-implantation mouse embryo. Mol. Syst. Biol..

[78] Maherali N., Sridharan R., Xie W., Utikal J., Eminli S., Arnold K. (2007). Directly reprogrammed fibroblasts show global epigenetic remodeling and widespread tissue contribution. Cell Stem Cell.

[79] Papp B., Plath K. (2011). Reprogramming to pluripotency: stepwise resetting of the epigenetic landscape. Cell Res..

[80] Singer M., Egloff F.R. (1949). The nervous system and regeneration of the forelimb of adult Triturus; the effect of limited nerve quantities on regeneration. J. Exp. Zool.

[81] Salpeter M.M. (1965). Disposition of nerve fibers in the regenerating limb of the adult newt, Triturus. J. Morphol..

[82] Singer M., Inoue S. (1964). The nerve and the epidermal apical cap in regeneration of the forelimb of adult triturus. J. Exp. Zool.

[83] Todd J.T. (1823). On the process of reproduction of the members of the aquatic salamander. Q. J. Sci. Lit. Arts.

[84] Endo T., Bryant S.V., Gardiner D.M. (2004). A stepwise model system for limb regeneration. Dev. Biol..

[85] Kawakami Y., Esteban C.R., Matsui T., Rodríguez-León J., Kato S., Izpisúa Belmonte J.C. (2004). Sp8 and Sp9, two closely related buttonhead-like transcription factors, regulate Fgf8 expression and limb outgrowth in vertebrate embryos. Development.

[86] Satoh A., Graham G.M., Bryant S.V., Gardiner D.M. (2008). Neurotrophic regulation of epidermal dedifferentiation during wound healing and limb regeneration in the axolotl (Ambystoma mexicanum). Dev. Biol..

[87] Makanae A., Mitogawa K., Satoh A. (2014). Co-operative Bmp- and Fgf-signaling inputs convert skin wound healing to limb formation in urodele amphibians. Dev. Biol..

[88] Satoh A., Makanae A., Nishimoto Y., Mitogawa K. (2016). FGF and BMP derived from dorsal root ganglia regulate blastema induction in limb regeneration in Ambystoma mexicanum. Dev. Biol..

[89] Mullen L.M., Bryant S.V., Torok M.A., Blumberg B., Gardiner D.M. (1996). Nerve dependency of regeneration: the role of distal-less and FGF signaling in amphibian limb regeneration. Development.

[90] Makanae A., Hirata A., Honjo Y., Mitogawa K., Satoh A. (2013). Nerve independent limb induction in axolotls. Dev. Biol..

[91] Thornton C.S., Abercrombie M., Brachet J., King T.J. (1968). Advances in Morphogenesis.

[92] Aguilar C., Gardiner D.M. (2015). DNA methylation dynamics regulate the formation of a regenerative wound epithelium during axolotl limb regeneration. PLoS One.

[93] Moore L.D., Le T., Fan G. (2013). DNA methylation and its basic function. Neuropsychopharmacology.

[94] Gallinari P., Di Marco S., Jones P., Pallaoro M., Steinkühler C. (2007). HDACs, histone deacetylation and gene transcription: from molecular biology to cancer therapeutics. Cell Res..

[95] Wang M.H., Hsu C.L., Wu C.H., Chiou L.L., Tsai Y.T., Lee H.S. (2021). Timing does matter: nerve-mediated HDAC1 paces the temporal expression of morphogenic genes during axolotl limb regeneration. Front. Cell Dev. Biol..

[96] Wang M.H., Wu C.H., Huang T.Y., Sung H.W., Chiou L.L., Lin S.P. (2019). Nerve-mediated expression of histone deacetylases regulates limb regeneration in axolotls. Dev. Biol..

[97] Voss S.R., Ponomareva L.V., Dwaraka V.B., Pardue K.E., Baddar N., Rodgers A.K. (2019). HDAC regulates transcription at the outset of axolotl tail regeneration. Sci. Rep..

[98] Taylor A.J., Beck C.W. (2012). Histone deacetylases are required for amphibian tail and limb regeneration but not development. Mech. Dev..

[99] Tseng A.S., Carneiro K., Lemire J.M., Levin M. (2011). HDAC activity is required during Xenopus tail regeneration. PLoS One.

[100] Johnson K., Bateman J., DiTommaso T., Wong A.Y., Whited J.L. (2018). Systemic cell cycle activation is induced following complex tissue injury in axolotl. Dev. Biol..

[101] Tanaka E.M., Drechsel D.N., Brockes J.P. (1999). Thrombin regulates S-phase re-entry by cultured newt myotubes. Curr. Biol..

[102] Ghosh S., Roy S., Séguin C., Bryant S.V., Gardiner D.M. (2008). Analysis of the expression and function of Wnt-5a and Wnt-5b in developing and regenerating axolotl (Ambystoma mexicanum) limbs. Dev. Growth Differ..

[103] Hayashi S., Tamura K., Yokoyama H. (2014). Yap1, transcription regulator in the Hippo signaling pathway, is required for Xenopus limb bud regeneration. Dev. Biol..

[104] Muneoka K., Holler-Dinsmore G.V., Bryant S.V. (1985). A quantitative analysis of regeneration from chimaeric limb stumps in the axolotl. J. Embryol. Exp. Morphol..

[105] Khattak S., Schuez M., Richter T., Knapp D., Haigo S.L., Sandoval-Guzmán T. (2013). Germline transgenic methods for tracking cells and testing gene function during regeneration in the axolotl. Stem Cell Rep..

[106] Monaghan J.R., Maden M. (2012). Visualization of retinoic acid signaling in transgenic axolotls during limb development and regeneration. Dev. Biol..

[107] Flowers G.P., Timberlake A.T., McLean K.C., Monaghan J.R., Crews C.M. (2014). Highly efficient targeted mutagenesis in axolotl using Cas9 RNA-guided nuclease. Development.

[108] Kragl M., Knapp D., Nacu E., Khattak S., Maden M., Epperlein H.H. (2009). Cells keep a memory of their tissue origin during axolotl limb regeneration. Nature.

[109] Moss F.P., Leblond C.P. (1970). Nature of dividing nuclei in skeletal muscle of growing rats. J. Cell Biol..

[110] Reznik M. (1969). Thymidine-3H uptake by satellite cells of regenerating skeletal muscle. J. Cell Biol..

[111] Snow M.H. (1977). Myogenic cell formation in regenerating rat skeletal muscle injured by mincing. II. An autoradiographic study. Anat. Rec..

[112] Tanaka H.V., Ng N.C.Y., Yang Yu Z., Casco-Robles M.M., Maruo F., Tsonis P.A. (2016). A developmentally regulated switch from stem cells to dedifferentiation for limb muscle regeneration in newts. Nat. Commun..

[113] Sandoval-Guzmán T., Wang H., Khattak S., Schuez M., Roensch K., Nacu E. (2014). Fundamental differences in dedifferentiation and stem cell recruitment during skeletal muscle regeneration in two salamander species. Cell Stem Cell.

[114] McCusker C.D., Diaz-Castillo C., Sosnik J., A Q.P., Gardiner D.M. (2016). Cartilage and bone cells do not participate in skeletal regeneration in Ambystoma mexicanum limbs. Dev. Biol..

[115] Mittenthal J.E. (1981). The rule of normal neighbors: a hypothesis for morphogenetic pattern regulation. Dev. Biol..

[116] Muneoka K., Fox W.F., Bryant S.V. (1986). Cellular contribution from dermis and cartilage to the regenerating limb blastema in axolotls. Dev. Biol..

[117] McCusker C.D., Gardiner D.M. (2013). Positional information is reprogrammed in blastema cells of the regenerating limb of the axolotl (Ambystoma mexicanum). PLoS One.

[118] Nacu E., Glausch M., Le H.Q., Damanik F.F., Schuez M., Knapp D. (2013). Connective tissue cells, but not muscle cells, are involved in establishing the proximo-distal outcome of limb regeneration in the axolotl. Development.

[119] Crawford K., Stocum D.L. (1988). Retinoic acid coordinately proximalizes regenerate pattern and blastema differential affinity in axolotl limbs. Development.

[120] Niazi I.A., Saxena S. (1978). Abnormal hind limb regeneration in tadpoles of the toad, Bufo andersoni, exposed to excess vitamin A. Folia Biol. (Krakow).

[121] Maden M. (1982). Vitamin A and pattern formation in the regenerating limb. Nature.

[122] Maden M. (1983). The effect of vitamin A on the regenerating axolotl limb. J. Embryol. Exp. Morphol..

[123] Nguyen M., Singhal P., Piet J.W., Shefelbine S.J., Maden M., Voss S.R. (2017). Retinoic acid receptor regulation of epimorphic and homeostatic regeneration in the axolotl. Development.

[124] Mercader N., Leonardo E., Piedra M.E., Martínez A.C., Ros M.A., Torres M. (2000). Opposing RA and FGF signals control proximodistal vertebrate limb development through regulation of Meis genes. Development.

[125] Mercader N., Tanaka E.M., Torres M. (2005). Proximodistal identity during vertebrate limb regeneration is regulated by Meis homeodomain proteins. Development.

[126] Roselló-Díez A., Ros M.A., Torres M. (2011). Diffusible signals, not autonomous mechanisms, determine the main proximodistal limb subdivision. Science.

[127] Selleri L., Depew M.J., Jacobs Y., Chanda S.K., Tsang K.Y., Cheah K.S. (2001). Requirement for Pbx1 in skeletal patterning and programming chondrocyte proliferation and differentiation. Development.

[128] Tzchori I., Day T.F., Carolan P.J., Zhao Y., Wassif C.A., Li L. (2009). LIM homeobox transcription factors integrate signaling events that control three-dimensional limb patterning and growth. Development.

[129] Wang Y.H., Beck C.W. (2014). Distal expression of sprouty (spry) genes during Xenopus laevis limb development and regeneration. Gene Expr. Patterns.

[130] Iwata R., Makanae A., Satoh A. (2020). Stability and plasticity of positional memory during limb regeneration in Ambystoma mexicanum. Dev. Dyn..

[131] Tabin C., Wolpert L. (2007). Rethinking the proximodistal axis of the vertebrate limb in the molecular era. Genes Dev..

[132] Suzuki M., Yakushiji N., Nakada Y., Satoh A., Ide H., Tamura K. (2006). Limb regeneration in Xenopus laevis froglet. Sci.WorldJournal.

[133] Hayashi S., Kawaguchi A., Uchiyama I., Kawasumi-Kita A., Kobayashi T., Nishide H. (2015). Epigenetic modification maintains intrinsic limb-cell identity in Xenopus limb bud regeneration. Dev. Biol..

[134] Wei X., Li H., Guo Y., Zhao X., Liu Y., Zou X. (2021). An ATAC-seq Dataset uncovers the regulatory landscape during axolotl limb regeneration. Front. Cell Dev. Biol..

[135] Baranowitz S.A., Maderson P.F., Connelly T.G. (1979). Lizard and newt tail regeneration: a quantitative study. J. Exp. Zool.

[136] Ghosh S., Thorogood P., Ferretti P. (1994). Regenerative capability of upper and lower jaws in the newt. Int. J. Dev. Biol..

[137] Reyer R.W. (1954). Regeneration of the lens in the amphibian eye. Q. Rev. Biol..

[138] Fernando W.A., Leininger E., Simkin J., Li N., Malcom C.A., Sathyamoorthi S. (2011). Wound healing and blastema formation in regenerating digit tips of adult mice. Dev. Biol..

[139] Simkin J., Sammarco M.C., Dawson L.A., Tucker C., Taylor L.J., Van Meter K. (2015). Epidermal closure regulates histolysis during mammalian (Mus) digit regeneration. Regeneration (Oxf).

[140] Lehoczky J.A., Robert B., Tabin C.J. (2011). Mouse digit tip regeneration is mediated by fate-restricted progenitor cells. Proc. Natl. Acad. Sci. U. S. A..

[141] Rinkevich Y., Lindau P., Ueno H., Longaker M.T., Weissman I.L. (2011). Germ-layer and lineage-restricted stem/progenitors regenerate the mouse digit tip. Nature.

[142] Mohammad K.S., Neufeld D.A. (2000). Denervation retards but does not prevent toetip regeneration. Wound Repair Regen..

[143] Atsuta Y., Lee C., Rodrigues A.R., Colle C., Tomizawa R.R., Lujan E.G. (2021). Direct reprogramming of non-limb fibroblasts to cells with properties of limb progenitors. bioRxiv.

[144] Chen Y., Xu H., Lin G. (2017). Generation of iPSC-derived limb progenitor-like cells for stimulating phalange regeneration in the adult mouse. Cell Discov..

[145] Mori S., Sakakura E., Tsunekawa Y., Hagiwara M., Suzuki T., Eiraku M. (2019). Self-organized formation of developing appendages from murine pluripotent stem cells. Nat. Commun..

[146] Yamada D., Nakamura M., Takao T., Takihira S., Yoshida A., Kawai S. (2021). Induction and expansion of human PRRX1(+) limb-bud-like mesenchymal cells from pluripotent stem cells. Nat. Biomed. Eng..

[147] McCusker C., Bryant S.V., Gardiner D.M. (2015). The axolotl limb blastema: cellular and molecular mechanisms driving blastema formation and limb regeneration in tetrapods. Regeneration (Oxf).

